# Identification of a truncated nucleoprotein in avian metapneumovirus-infected cells encoded by a second AUG, in-frame to the full-length gene

**DOI:** 10.1186/1743-422X-2-31

**Published:** 2005-04-12

**Authors:** Rene Alvarez, Bruce S Seal

**Affiliations:** 1 Southeast Poultry Research Laboratory, Agricultural Research Service, U.S. Department of Agriculture, Athens, GA 30605, USA; 2 Present address: Department of Infectious Diseases, College of Veterinary Medicine, University of Georgia, Athens, GA 30605, USA; 3 Poultry Microbiological Safety Research Unit, ARS, USDA, 950 College Station Rd., Athens, GA 30605, USA

## Abstract

**Background:**

Avian metapneumoviruses (aMPV) cause an upper respiratory disease with low mortality, but high morbidity primarily in commercial turkeys. There are three types of aMPV (A, B, C) of which the C type is found only in the United States. Viruses related to aMPV include human, bovine, ovine, and caprine respiratory syncytial viruses and pneumonia virus of mice, as well as the recently identified human metapneumovirus (hMPV). The aMPV and hMPV have become the type viruses of a new genus within the *Metapneumovirus*. The aMPV nucleoprotein (N) amino acid sequences of serotypes A, B, and C were aligned for comparative analysis. Based on predicted antigenicity of consensus protein sequences, five aMPV-specific N peptides were synthesized for development of peptide-antigens and antisera.

**Results:**

The presence of two aMPV nucleoprotein (N) gene encoded polypeptides was detected in aMPV/C/US/Co and aMPV/A/UK/3b infected Vero cells. Nucleoprotein 1 (N1) encoded from the first open reading frame (ORF) was predicted to be 394 amino acids in length for aMPV/C/US/Co and 391 amino acids in length for aMPV/A/UK/3b with approximate molecular weights of 43.3 kilodaltons and 42.7 kilodaltons, respectively. Nucleoprotein 2 (N2) was hypothesized to be encoded by a second downstream ORF in-frame with ORF1 and encoded a protein predicted to contain 328 amino acids for aMPV/C/US/Co or 259 amino acids for aMPV/A/UK/3b with approximate molecular weights of 36 kilodaltons and 28.3 kilodaltons, respectively. Peptide antibodies to the N-terminal and C-terminal portions of the aMPV N protein confirmed presence of these products in both aMPV/C/US/Co- and aMPV/A/UK/3b-infected Vero cells. N1 and N2 for aMPV/C/US/Co ORFs were molecularly cloned and expressed in Vero cells utilizing eukaryotic expression vectors to confirm identity of the aMPV encoded proteins.

**Conclusion:**

This is the first reported identification of potential, accessory in-frame N2 ORF gene products among members of the *Paramyxoviridae*. Genomic sequence analyses of related members of the *Pneumovirinae *other than aMPV, including human respiratory syncytial virus and bovine respiratory syncytial virus demonstrated the presence of this second potential ORF among these agents.

## Background

Avian metapneumovirus (aMPV) causes turkey rhinotracheitis (TRT) and is associated with swollen head syndrome (SHS) of chickens that is usually accompanied by secondary bacterial infections which can increase morbidity and induce mortality. Avian metpnuemovirus was first reported in South Africa during the early 1970s and was subsequently isolated in Europe, Israel and Asia [1,2]. During 1997, mortality due to aMPV infections among commercial turkeys in the U.S. ranged from zero, to 30% when accompanied by bacterial infections, with condemnations due to air sacculitis. This was the first reported outbreak of aMPV infections in the U.S. which was previously considered exotic to North America. The virus causing disease was designated a new aMPV type C genetically different from European counterparts [3-5] and was subsequently demonstrated to be most closely related to human metapneumovirus (hMPV) from diverse geographic locations [6,7]. Infections among commercial turkeys with aMPV/C continue in the north-central U.S. resulting in substantial economic loss to the poultry industry [6,8,9].

Pneumoviruses are members of the family *Paramyxoviridae *that contain a nonsegmented, negative-sense RNA genome of approximately 15 kb in length. Viruses related to aMPV include human, bovine, ovine and caprine respiratory syncytial viruses and pneumonia virus of mice [10], as well as the recently identified hMPV [11]. Although genome length is similar, pneumoviruses generally encode ten genes, compared to six or seven in other paramyxoviruses. These include the nonstructural proteins (NS1 and NS2), nucleoprotein (N), phosphoprotein (P), matrix protein (M), small hydrophobic protein (SH), surface glycoprotein (G), fusion protein (F), second matrix protein (M2) and a viral RNA-dependent RNA polymerase (L). The pneumoviruses have an F protein that promotes cell fusion, but these viruses do not hemagglutinate, nor do they have neuraminidase activity in their G attachment protein. This is an important distinguishing characteristic from the other paramyxoviruses [10].

Because of a limited genome size, many non-segmented RNA viruses, including the pneumoviruses, have devised mechanism to increase protein coding capacities. This may occur at two levels: 1) transcriptional mRNA processing or modification [12-14] or 2) translational, in which proteins may be produced from alternative open reading frames (ORFs) or from translational initiation at non-AUG or downstream AUG codons [15-17]. Among the pneumoviruses, secondary coding usage has only been documented for the M2 gene, which encodes two proteins. The M2-1, a transcription antitermination factor, is required for processive RNA synthesis and transcription read-through at gene junctions. The M2-2 is involved with the shift between viral RNA transcription and replication [18]. In this report, we present evidence for utilization of a secondary open reading frame, within the N gene encoding a truncated nucleoprotein (N2) among aMPV/C/Co and aMPV/A/UK/3b infected cells.

## Results

### Avian metapneumovirus N gene possess several putative AUG start sites

**Figure 1:**
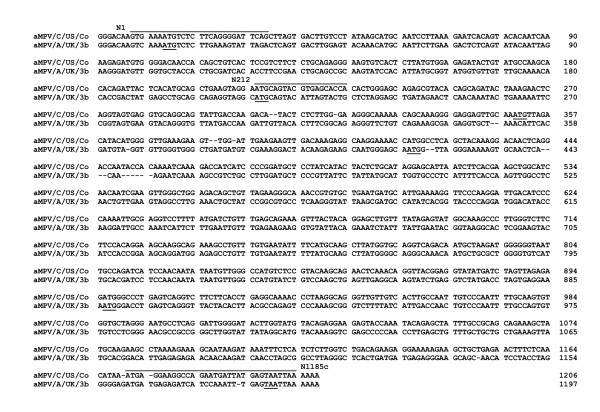
Alignment of avian metapneumovirus type A and C nucleoprotein genes demonstrating presence of multiple start sites. Underlined sequences denote hypothesized alternative in-frame start sites and the stop codon. Primer sequences utilized for cDNA synthesis of nucleoprotein genes are also illustrated.

The aMPV/C/US/Co nucleoprotein is encoded by the N gene with a predicted molecular weight of 42–45 kD [7,19]. The N gene ranges from 1191 to 1206 nucleotides in length [6,19], with the first AUG at nucleotide position 14 (Fig. 1) in all three subtypes (A, B, and C). The aMPV/C/US/Co N gene has additional putative start sites at nucleotide positions 212, 350, 416, 758, 785, 827, 896, and 1022 with "true" Kozak sequences [20] at nucleotide positions 413 (ACCAUGG) and 893 (GAGAUGG), with predicted translation products of 28.5 kD and 10.78 kD, respectively. The aMPV/A/UK/3b N gene has additional putative start sites at nucleotide positions 161, 212, 293, 410, 413, 605, 722, 749, 749, 776, 818, 887, and 1013 with "true" Kozak sequences [20] at nucleotide positions 602 (AGGAUGG), 719 (AGGAUGG), and 884 (AAAAUGG), with predicted translation products of 21.26 kD, 16.73 kD, and 10.54 kD, respectively.

### Avian metapneumovirus-infected cells produce two proteins (N1 and N2) encoded by two open-reading frames within the N gene

**Figure 2:**
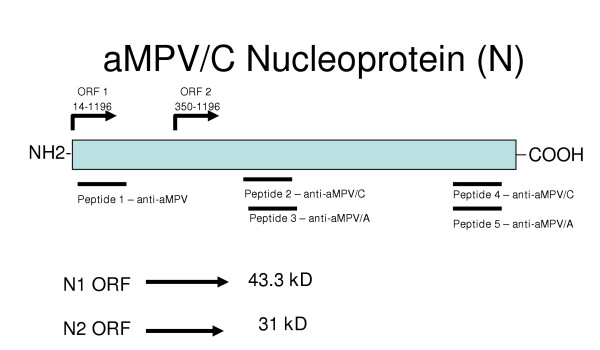
Relative position of peptides within the avian metapneumovirus nucleoproteins utilized for generation of affinity purified polyclonal antibodies.

**Figure 3:**
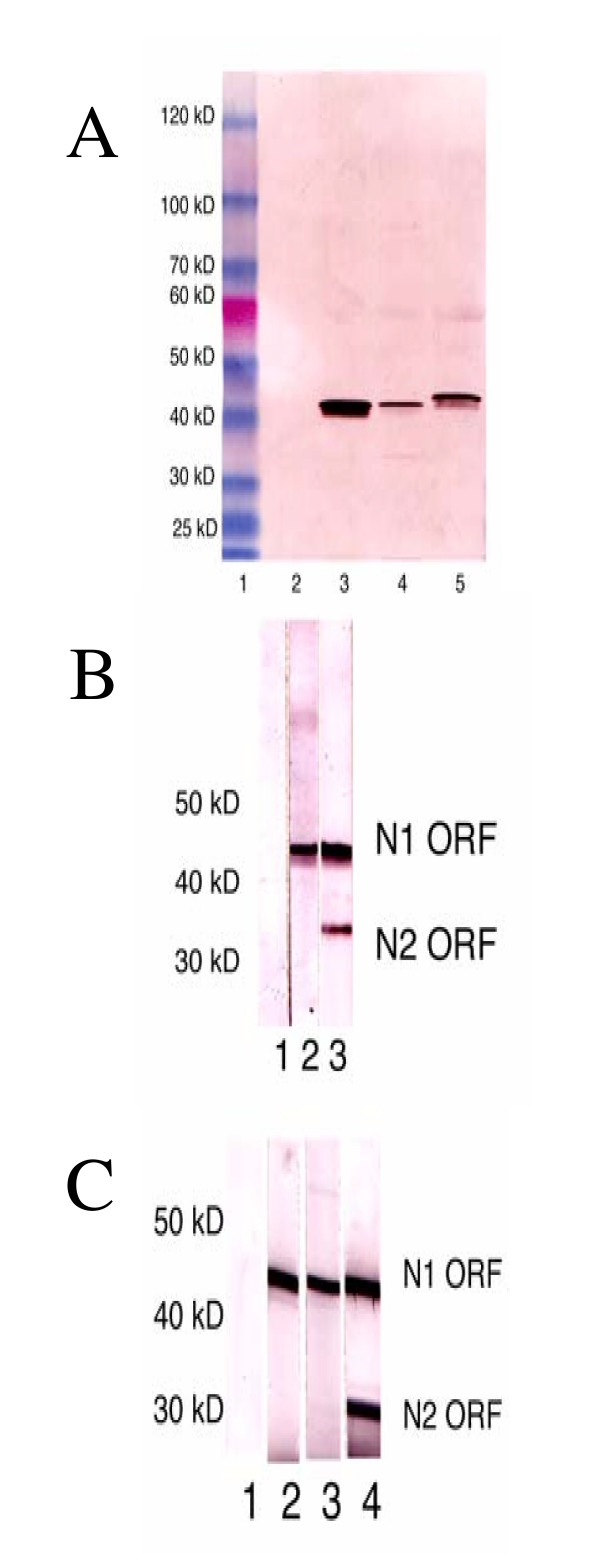
Detection of avian metapneumovirus (aMPV) nucleoprotein gene products among infected cells utilizing affinity purified peptide antibodies. A. Antibody reacted against an N-terminal portion of the nucleoprotein designed to detect all aMPV serotypes N1. Lane 1: molecular size markers; Lane 2: uninfected cell proteins; Lane 3: aMPV/A infected cell proteins; Lane 4: aMPV/B infected cell proteins; Lane 5: aMPV/C infected cell proteins. B. Antibody detection of a C-terminal portion of the aMPV/C nucleoprotein. Lane 1: uninfected cell proteins; Lane 2: aMPV/C infected cell proteins reacted with N1 peptide antibodies; Lane 3: aMPV/C infected cells reacted with aMPV/C-specific N2 peptide antibodies. C. Antibody detection of a C-terminal portion of the aMPV/A nucleoprotein. Lane 1: uninfected cell proteins; Lane 2: aMPV/A infected cell proteins reacted with N1 peptide antibodies; Lane 3: aMPV/A infected cell proteins reacted with N3 peptide antibodies; Lane 4: aMPV/A infected cells reacted with N5 peptide antibodies.

Five peptides within the aMPV N gene (Fig. 2) were utilized to generate affinity-purified rabbit peptide antibodies. This approach was exploited to determine if any of the alternative start sites of the aMPV N gene were utilized during an active cell infection. aMPV/N-peptide antibody directed against aMPV/C/US/Co N protein amino acids 10–29 (DLSYKHAILKESQYTIKRDV) with only 3 changes in both aMPV types A and B at amino acid positions 12 (S to E), 19 (K to D) and 26 (K to R) reacted with all three full length nucleoproteins by western blot (Fig. 3A, Lanes 3, 4, and 5), but did not react with any proteins in uninfected Vero cells (Fig. 3A, Lane 2). All three virus nucleoproteins were between 42–45 kD based on SDS-PAGE/western blot analysis (Fig. 3A). We then tested the aMPV/C-N2 peptide antibody directed against amino acids 128–148 in the mid-portion of the of the aMPV/C/US/Co isolate (Fig. 2) by western blot which would recognize any downstream translation products encoded by the N gene and utilization of any secondary start sites. Western blot analysis revealed two putative N gene products in aMPV/C/US/Co-infected Vero cells, the first, the full-length nucleoprotein with a molecular weight of approximately 43 kD (Fig. 3B, Lane 3) and the second, a smaller protein of approximately 35–36 kD (Fig. 3B, Lane 3). The peptide antibody to amino acids 303 to 393 (aMPV/C-N4) synthesized to be reactive to the C-terminal N protein from aMPV/C also recognized two proteins as in Fig. 3B, Lane 3 (data not shown).

To evaluate whether the utilization of alternative start sites was unique to members of the aMPV type C group, or whether this also occurred in other aMPV types, we utilized aMPV/A-N3 and aMPV/A-N5 peptide antibodies (anti-aMPV/Type A, N protein, amino acids 126–145 and 380–390, respectively). Unlike aMPV/C-N2 peptide antibody, aMPV/A-N3-peptide antibody (amino acids 126–145) reacted to only a full length nucleoprotein (Fig. 3C, lane 3) similar to the aMPV/N-peptide antibody (Fig. 3C, lane 2), while aMPV/A-N5-peptide antibody (amino acids 380–390) reacted with both the full length nucleoprotein of approximately 41–43 kD (Fig. 3C, lane 4) and a smaller protein of approximately 28–30 kD (Fig. 3C, lane 4). Finally, all aMPV type-specific antibodies were not cross active with other metapneumoviruses (data not shown).

### Expression of the N1 and N2 ORF of avian metapneumovirus type C/Colorado in eukaryotic cells

Sequence analysis of the aMPV/C/US/Co and aMPV/A/UK/3b N gene nucleotide sequences revealed that downstream of the first AUG (position 14) were multiple putative start sites as described above (Fig. 1). We therefore utilized sequence analysis software to analyze the N gene putative open reading frames and the predicted translation products from each putative start site for products that would result in proteins of approximate size as the smaller reactive band that was detected by western blot (Fig. 3B, lane 3 and Fig. 3C, lane 4). Two predicted proteins in the aMPV/C/US/Co sequences corresponding to a predicted molecular weight of approximately 31.12 kD (third AUG) and another at 28.5 kD (fourth AUG) were detected in the N gene sequence.

**Figure 4:**
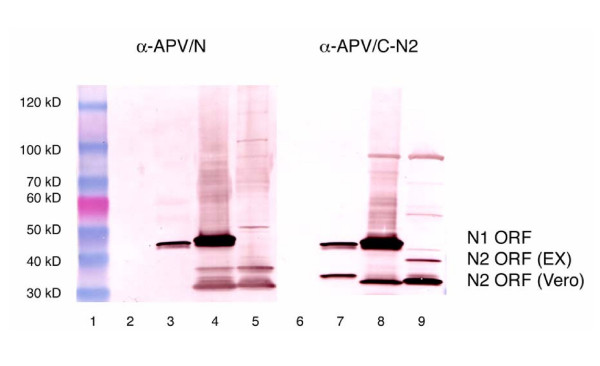
Expression of N1 and N2 open reading frames of avian metapneumovirus type C in transfected eukaryotic cells by an expression vector. Lane 1: molecular size markers; Lane 2: uninfected control cells; Lane 3. aMPV/C infected cells reacted with antibodies to peptide N1. Lane 4: Cells transformed with aMPV/C-N gene complete ORF reacted with antibodies to peptide N1. Lane 5: Cells transformed with expression plasmid with truncated N2ORF reacted to antibodies to peptide N1; Lane 6: uninfected control cells; Lane 7: aMPV/C infected cells reacted to antibodies to peptide N4. Lane 8: Cells transformed with aMPV/C-N gene complete ORF reacted with antibodies to peptide N2. Lane 9: Cells transformed with expression plasmid with truncated N2ORF reacted to antibodies to peptide N2.

Since SDS-PAGE analysis is not necessarily an accurate measurement of molecular size, both starts sites could result in a protein observed at approximately 35–36 kD by SDS-PAGE, and therefore either site could result in the second ORF product. We therefore used two primer sets N1/N1189C and N212/N1189C which spans either the full length of ORF1 or the ORF2 and any down stream putative ORFs of aMPV/C/US/Co, respectively (Fig. 2) to amplify both ORFs by RT-PCR. Both ORFs were amplified and cloned into a eukaryotic expression vector. Western blot analysis of the Vero cell expressed N1 and N2 ORFs revealed one reactive band in the pCR3.1-N1ORF transfected Vero cells with the aMPV/N antibody (Fig. 4, lane 4) corresponding to the full length nucleoprotein of aMPV, similar to that observed in aMPV-infected Vero cells (Fig. 4, lane 3). This protein was not visualized in the pCR3.1-N2ORF transfected Vero cells (Fig. 4, lane 5), as was expected since the N212 primer is downstream of the peptide (aMPV/N, amino acids, 10–29) utilized to synthesize aMPV/N peptide antibody. However, when the aMPV/C-N2 (peptide antibody directed to amino acids 383–393 of aMPV/C N protein) was used for western blot analysis, two proteins were reactive in the pCR3.1-N1ORF Vero cells, the first at approximately 43 kD (Fig. 4, lane 8), similar to that observed in aMPV-infected Vero cells (Fig. 4, Lane 7) and the second, a protein of approximately 35 kD (Fig. 4, Lane 8), slightly smaller than the N2 ORF protein in aMPV-infected Vero cells (Fig. 4, Lane 7). Western blot analysis of the pCR3.1-N2ORF induced Vero cells demonstrated one reactive band of approximately 35 kD (Fig. 4, Lane 9), similar to the smaller reactive band in the pCR3.1-N1ORF transfected Vero cells. The full-length nucleoprotein, as expected was not present in the pCR3.1-N2ORF transfected Vero cells, since the N212 primer is downstream of the first AUG start site (position 14).

## Discussion

The utilization of alternative open reading frames for the expansion of genetic information in negative-stranded RNA viruses has been well documented [10,16,17,21,22]. There are, however, various mechanisms for accessing this genetic information. The phosphoprotein of measles virus encodes a single mRNA, which is read in two independently initiated overlapping reading frames [17], while transcripts of influenza virus gene segments 7 and 8 are spliced within the nucleus for production of two different sizes of mRNAs sharing the same 5'-proximal AUG initial codon [16]. The P gene of Sendai virus is reported to be transcribed into two polycistronic mRNAs, P/C and V/C, which are translated to synthesis the P, C, C', Y1, and Y2 proteins from independent start sites in two overlapping reading frames [23-25].

Within the *Paramyxoviridae*, Newcastle disease virus possesses a polycistronic phosphoprotein (P) gene. Transcriptional modification of the NDV P gene mRNA allows for potential expression of two smaller putative proteins, designated V and W [12], that appears to be a result of polymerase stuttering at the editing site sequences [13,14], leading to the insertion of non-template G nucleotides within the P gene [12]. Consequently, during translation there is a frame shift resulting in production of the V or W protein, dependent on the number of G nucleotides inserted [12]. It was previously suggested that NDV [26] potentially utilized an alternative in-frame AUG start site for expression of an accessory protein similar to the Sendai virus X protein [21] that was recently demonstrated to not be utilized during infection of cells in culture [27].

Pneumonia virus of mice, human and bovine respiratory syncytial viruses, and avian metapneumovirus also possess polycistronic gene(s) [28-30]. The M2 gene of all the pneumoviruses contains two partially overlapping open reading frames, with the 5'-proximal open reading frame favored for utilization by the criteria of location and sequence of its start site [28,29]. The P gene of the pneumonia virus of mice is the only known polycistronic phophoprotein gene in the pneumoviruses, and utilizes internal initiation of in-frame AUG initiation codons to generate up to four additional carboxy co-terminal products [30].

In this present study, we demonstrated that the nucleoprotein gene of the avian metapneumovirus subtypes A and C are putatively polycistronic. This may occur by utilization of a second in-frame initiation site (AUG) for the generation of a truncated nucleoprotein present among infected Vero cells. Sequence analysis demonstrated the presence of multiple putative initiation (AUG) start sites along the N gene, however only one alternative start site at nucleotide positions 212 and 410 for APV/C and APV/A, respectively appear to be utilized to transcribe the N2 protein seen in infected cells.

The N protein of Pneumoviruses ranges in size from 42–45 kD, based on SDS-PAGE relative mobility, and is highly conserved among metapneumoviruses [7]. The N protein, which protects the RNA genome from ribonucleases, is associated with other viral proteins (P, M2, and L), which together form the transcription complex. The nucleocaspid is the template for transcription and replication; the RNA genome by itself cannot fulfill the role of template. Pneumovirus infection in cells results in the accumulation of the N protein in cytoplasmic inclusion bodies that can be visualized by immunofluorescence [31] or immunohistochemistry [7] as relatively large dots that are usually close to the nucleus of infected cells.

Mapping of several paramyxovirus N proteins, including Sendai and measles virus, indicated that the N protein has two major domains; the amino terminal domain appears to be required for nucleoprotein formation, containing the domains necessary for RNA binding and N-N interactions; while the carboxy-domain interacts with the phosphoprotein (P), particularly when it is part of the polymerase complex [32,33]. In bovine respiratory syncytial virus (bRSV), removal of the C-terminal 32 amino acids of the N protein inhibits the interactions with the P protein, whereas the removal of 32 amino acids from the N-terminus has a minimal effect [32]. However, almost all of the N from amino acids 2–391 is required to support bRSV minigenome RNA synthesis [34]. The truncated N2 protein encompasses 328 amino acids (250 for aMPV/Type A) of the carboxy terminus of the full-length N protein, suggesting that N2 may not be involved in the polymerase complex. However the domains responsible for RNA binding of N-N and N-P binding remain intact, suggesting that N2 may play an alternative role in cells during viral infection.

## Methods

### Cells and viruses

Vero cells were maintained as monolayer cultures in minimal essential media (MEM) supplemented to contain 8 % fetal bovine serum with 100 units/ml penicillin G, 0.025 μg/ml amphotericin B, and 100 units/ml streptomycin. The aMPV/C/US/Co and aMPV/A/UK/3b isolates were obtained from the National Veterinary Services Laboratory (NVSL, APHIS, USDA, Ames, Iowa). Viruses were propagated on 95% confluent Vero cell monolayers in MEM supplemented to contain 2% FBS and antibiotics as described previously [3]. Cells were infected at multiplicity of infection of 10 (moi = 10), and virus was adsorbed for 1 hour at 37°C. Media was added and cells were incubated at 37°C, 5% CO_2 _for 72 hours or until 90% cytopathic effect was observed by light microscopy. Cells were scraped and harvested by centrifugation at 8000 × g.

### Computer analyses, peptide synthesis and antibody production

The nucleoprotein (N) gene sequences of aMPV serotypes A, B, and C (Genbank accession numbers: AAC55065, AAG42499, and AAF05909) were analyzed in the GeneWorks (Intelligentics, Mountain View, CA) and Mac Vector (Accelrys, San Diego, CA) computer analysis programs to determine hydrophilicity, antigenicity, and identity of the deduced amino acid sequences. The sequences were aligned for maximum similarity, and a consensus sequence was determined using the most prevalent amino acid for each residue. Five peptides with sequences: 1) aMPV/N: DLSYKHAILKESQYTIKRDV; 2) aMPV/C-N2: DKEARKTMASATKDNSGPIPQ; 3) aMPV/A-N3: ERTTREAMGAMVREKVQLTK; 4) aMPV/C-N4: LNINEEGQNDY; and 5) aMPV/A-N5: LGGDDERSSKF were chosen based on antigenicity and hydrophilicity to be utilized for generation of aMPV peptide-based antibodies. Peptides were synthesized by Research Genetics (Huntsville, AL) according to the manufacturer's protocol.

Briefly, rabbit aMPV/N peptide antibodies were produced by Research Genetics (Huntsville, AL) according to manufacturer's protocol. Two rabbits were injected with 0.1 mg of KLH-conjugated peptide emulsified with Freud's complete adjuvant and injected into four subcutaneous (SQ) sites on day 1. On days 14, 42, and 56 rabbits were injected again (boosters) with 0.1 mg of KLH-conjugated peptide emulsified with Freud's complete adjuvant [35]. Sera were collected at days 0, 28, 56 and 70. Rabbit pre-immune sera were used as negative controls for rabbit assays.

### SDS-PAGE and Western blot assay

Protein concentration of the supernatant fraction from infected cells was measured for protein concentration by Bradford's reagent (Bioworld, Dublin, OH) at 595 nm. Infected supernatants were denatured in Laemmli's sample buffer (BioRad, Hercules, CA) and boiled for 5 min. Denatured polypeptides (6 μg protein/lane) were separated in a sodium dodecyl sulfate 4–20% polyacrylamide Criterion (Biorad, Hercules, CA) gel gradient by electrophoresis (SDS-PAGE) at 120 V for 2 hours [36]. Polypeptides were transferred to nitrocellulose by applying a constant voltage of 10 V for 1 hour on a Biorad (Hercules, CA) Trans-Blot SD Semi-Dry Transfer cell [37]. Blots were blocked with BLOTTO (20% dry milk in PBS) overnight at 4°C or for 1 hour at 37° and washed 3 X in phosphate buffered saline (PBS). Affinity purified rabbit anti-peptide antibody (diluted 1:100) was used as the source of the primary antibodies and incubated for 1 hour at 37°C followed by 3 washes in PBS. Secondary antibody (α-rabbit IgG-alkaline phosphatase, Sigma, The Woodlands, TX) was added (1:500), incubated 1 hour at 37°C, washed 3 X in PBS and developed using a alkaline phosphatase substrate kit (Vector, Burlingame, CA).

### Viral RNA Isolation accompanied by RT-PCR Amplification of aMPV/C/US/Co N1 and N2 ORF nucleotide sequences

Total RNA was isolated [38] from aMPV/C/US/Co-infected Vero cell lysates using Qiagen's "RNeasy" kit (Qiagne, Valencia, CA) according to the manufacturer's protocol. RNA was analyzed for purity by agarose gel electrophoresis in a 1.5% agarose gel, at 125 volts, and stained with 10 μg/ml of ethidium bromide (Sigma, The Woodlands, TX). The aMPV N1 and N2 ORFs were reverse transcribed using either the N1 (5'-GAAATGTCTCTTCAGGGGATTCAG-3') and N1185C (5'-AATCATTCTGGCCTTCCTCAT-3') primer pair or the N212 (5'-ATGCAGTACGTGAGCACC-3') and N1185C (5'-AATCATTCTGGCCTTCCTCAT-3') primer pair, followed by 30 cycles of PCR [39]. RT-PCR amplification products were analyzed by agarose gel electrophoresis and the full length N1 ORF product and the N2 ORF product were excised and purified before cloning into the expression vector pCR3.1-Topo (Invitrogen, Carlsbad, CA).

### Molecular cloning, nucleotide sequencing, and eukaryotic expression of pCR3.1-N1ORF and pCR3.1-N2ORF

The N1 ORF and N2 ORF fragments of aMPV/C/US/Co were cloned into the eukaryotic expression vector pCR3.1-Topo (Invitrogen, Carlsbad, CA) according to the manufacturer's protocol. Plasmid DNA was isolated using Qiagen's miniprep kit (Qiagen, Valencia, CA). Double stranded sequencing with *Taq *polymerase (Applied Biosystems Inc.) and fluorescent labeled dideoxynucleotides was performed with an automated sequencer [40] on both amplification products to verify identity and insure that no changes in the ORFs had been made relative to the original N gene. The pCR3.1-N1ORF and pCR3.1-N2ORF vectors were transfected into Vero cells using lipofectamine (Invitrogen, Carlsbad, CA). Protein was induced with IPTG (Sigma, The Woodlands, TX) at 24 hours post-transfected and total proteins were harvested by scraping. An aliquot of uninduced and induced cells were lysed in 2 X Laemmli's buffer, boiled for 5 minutes and separated by SDS-PAGE on a 4–20% Criterion (Biorad, Hercules, CA) gradient gel, followed by electroblotting onto nitrocellulose as previously described.

## Competing Interests

The author(s) declare that they have no competing interests.

## Authors' contributions

Dr. Alvarez was a post-doctoral associate and conducted the primary experimentation following design of peptides and production of anti-sera under the direction of Dr. Seal. Dr. Alvarez initiated writing of the draft manuscript with subsequent editing and revisions by both authors.
